# Assessing the cost of illness of RSV and non-RSV acute respiratory infections in Nepali children

**DOI:** 10.7189/jogh.15.04092

**Published:** 2025-04-11

**Authors:** Neele Rave, Arun K Sharma, Ram H Chapagain, Rupesh Shrestha, An Nguyen, Clint Pecenka, Farina L Shaaban, Prakash Joshi, Louis J Bont, RSV GOLD III – Health Economics Study Group, Fadlulai Abdu-Raheem, Anas Abubakar, Rosemary Akuaku, Abdullahi Aminu, Louis Bont, Ram H Chapagain, Assucênio Chissaque, Andrew Clark, Joycelyn Dame, Frédéric Debellut, Nilsa de Deus, Rita Dhital, Upendra Dhungana, Amma Ekem, Norbert Fuhngwa, Maria A Garba, Fatima J Giwa, Bamenla Goka, Esperança L Guimarães, Prakash Joshi, Ranju Karki, Habiba Lawal, Bernsah D Lawong, Braiton Maculuve, Henshaw Mandi, Elias Manjate, Yara Manjate, Izilda Matimbe, Abdullahi Musa, Tufária Mussá, Teddy Naddumba, Adita Nepali, An Nguyen, Ebenezer Ntow, Aira A Olorukooba, Kwabena A Osman, Mirela Pale, Cesar Palha, Uttam Paudel, Clint Pecenka, Neele Rave, Farina L Shaaban, Arun K Sharma, Rupesh Shrestha, Cristina Sinussene, Nirasta Thakili, Farida Zavala

**Affiliations:** 1 Department of Paediatrics, University Medical Centre Utrecht, Utrecht, The Netherlands; 2 Department of Paediatrics, Institute of Medicine, Tribhuvan University Teaching Hospital, Kathmandu, Nepal; 3 Department of Paediatrics, Kanti Children's Hospital, Kathmandu, Nepal; 4 PATH, Center for Vaccine Innovation and Access, Ho Chi Minh City, Vietnam; 5 PATH, Center for Vaccine Innovation and Access, Seattle, Washington, USA; 6 Nepal Paediatric Society, Kathmandu, Nepal

## Abstract

**Background:**

Low- and middle-income countries (LMICs) bear the greatest burden of the global respiratory syncytial virus (RSV) morbidity and mortality, but lack cost data to evaluate the health-economic impact of RSV burden on families, the healthcare system, and society. This prospective observational study was performed by the RSV GOLD III – Health Economic Research Group and estimated the costs associated with RSV illness in Nepal.

**Methods:**

We collected healthcare resource utilisation data from children <2 years old fulfilling the World Health Organization (severe) acute respiratory infections ((S)ARI) case definition over one local respiratory season (July to November 2023) at two public hospitals in Nepal. We used hospital records and caregiver interviews to collect direct medical, direct non-medical, and indirect cost data to generate total per-patient costs.

**Results:**

We included 730 patients with a mean age of 6.8 (standard deviation = 5.8) months. RSV infection was confirmed in 72.6% of the inpatients (n/N = 469/646) with SARI. The mean total cost per RSV episode was USD 43 (95% confidence interval (CI) = 25–62) for non-severe, USD 312 (95% CI = 293–332) for severe, and USD 664 (95% CI = 381–947) for life-threatened patients. Of the total costs, the healthcare system incurred USD 16 (36.3%), USD 58 (18.6%), and USD 57 (8.6%) in each category of illness. Household-level costs were 1.4% (USD 19) of the country’s gross domestic product per capita for non-severe, 15.1% (USD 200) for severe, and 35.7% (USD 472) for life-threatened patients, with costs for inpatients often reaching catastrophic levels.

**Conclusions:**

Our findings show a significant healthcare and economic burden of RSV illness in Nepal, highlighting the need to prioritise RSV prevention strategies. Our cost burden data can inform the modelling of costs and benefits of future RSV interventions in Nepal.

Respiratory syncytial virus (RSV) is an important cause of hospitalisation and mortality due to lower respiratory tract infections (LRTIs) in children under five years old worldwide. An estimated 33 million respiratory tract infections and 118 000 deaths are associated with RSV annually, with most infections and deaths occurring in low- and middle-income countries (LMICs) [1]. RSV tends to be most severe in young infants aged less than six months, with most infections occurring within the first year of life [1]. Global estimates suggest that RSV contributes approximately 1.2 million disability-adjusted life years, imposing an economic burden of approximately USD 5.4 billion annually [2]. A significant proportion of this burden is borne by LMICs, with nearly 55% of global costs attributed to acute lower respiratory tract infections requiring hospitalisation [2].

Acute respiratory infections (ARIs) persist as a considerable public health problem in Nepal, with a prevalence rate of 2.1% observed among children under five years old [3]. Data on the RSV disease burden and associated costs, however, remain sparse. The virus has been identified as a major primary respiratory viral pathogen in young Nepali children hospitalised with severe pneumonia, as well as in rural community settings [4]. Furthermore, recent evidence has shown its significant impact on the intensive care unit admissions in urban Nepal during the 2021 and 2022 seasons, where it was found to account for 27.9% of the children with severe ARI [5]. For this reason, RSV prevention strategies are needed to protect young infants who are at the highest risk of severe RSV-associated LRTIs in Nepal.

Several intervention strategies against RSV are now available that can potentially change this scenario, two of which have recently been market approved. A maternal prefusion RSV vaccine (RSVpreF) demonstrated promising results in a phase III maternal RSV immunisation trial, showing vaccine efficacy of 81.8% (99.5% confidence interval (CI) = 40.6–96.3) against severe medically attended lower respiratory tract infection (LRTI) due to RSV in infants from birth through the first 90 days of life [6]. Similarly, a long-acting monoclonal antibody (mAb), nirsevimab, administered as a single injection at birth, has demonstrated high efficacy (76–80%) in clinical trials against severe RSV-LRTI and medically attended RSV-LRTI up to 150 days in preterm and full-term infants [7-9]. Optimal implementation of these interventions in LMICs will require accurate studies of disease costs to inform cost-effectiveness analyses and guide decision-making.

Despite the substantial impact of RSV on public health and economics, data on its financial burden on families and the healthcare system remain limited in LMICs. Some studies, however, have provided insight into the economic impact in these settings. For instance, recent research in Kenya reported the mean costs of severe RSV illness among infants, expressed in 2021 USD, to be USD 329 (95% CI = 251–408) and USD 527 (95% CI = 405–649), respectively, in two semi-rural regions of Kenya [10]. Estimates from 2020 in Malawi reported mean costs per RSV episode at USD 62 (95% CI = 51–74) [11]. To our knowledge, the only recent study from an Asian LMIC comes from Vietnam; it estimated the median costs of RSV-associated LRTI at USD 165 (IQR = 95–249) [12]. Thus, cost estimates are needed to further understand the economic burden of RSV in LMICs, particularly in regions like Nepal.

We aimed to estimate the cost of RSV illness in Nepali children from the perspectives of the health system, households, and society. Our findings could provide valuable insights for informing decision-making regarding the cost-effectiveness of introducing RSV intervention strategies for improved health outcomes.

## METHODS

This prospective research project is part of a larger study, the RSV Global Online Mortality Database (GOLD) III – Health Economics Study, conducted in four LMICs: Ghana, Mozambique, Nepal, and Nigeria. The study took place during one local respiratory season to assess the cost of illness of RSV in children <2 years old. Its foundational approach has been described in detail elsewhere [13]. Here we provide an overview of the most critical elements and highlight any country-specific deviations.

### Study setting

This study took place at the outpatient and inpatient departments of the Kanti Children’s Hospital (KCH) and Tribhuvan University Teaching Hospital (TUTH) in Kathmandu, Nepal. The KCH is a referral paediatric hospital, while the TUTH is a tertiary referral general hospital. Each of the hospitals provides outpatient care six days a week, along with a general paediatric ward and high-dependency care that includes intensive care. The KCH has 108 beds in the general wards, 12 in the high-dependency unit (HDU), and 28 in the neonatal/paediatric intensive care unit (ICU). The TUTH has 54 general ward beds, 6 HDU beds, and 12 neonatal/paediatric ICU beds.

### Study population

All patients between four days to <2 years old admitted or visiting the outpatient department of either hospital between July and November 2023 that met the World Health Organization (WHO) (S)ARI case definition were eligible for enrolment, if parent/guardian consent was provided. The severity of RSV was classified on the location of admission: non-severe for outpatient visits only; severe for admission to the general, respiratory, and intermediate wards or HDU; and life-threatened for ICU admission or death.

Due to the high volumes of patients at the outpatient clinic and an inability to evaluate every patient with respiratory symptoms, only a subset of patients seen at the outpatient clinic were randomly included in the study whenever data collectors were available to screen patients in the clinic for acute respiratory symptoms.

The Nepal Health Research Council, ethical review committees of both hospitals, and the Medical Research Ethics Committee NedMec (#20–536) of the University Medical Centre in Utrecht approved the study protocol. All study procedures followed relevant local regulations and the principles of the Declaration of Helsinki.

### Sample size calculations

To estimate the number needed to achieve the desired sample sizes, we used the following formula [14]:



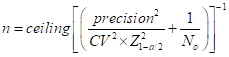



We utilised a Z-score of 1.96 (95% CI), a coefficient of variation of 0.5, and a precision level of ±10% of the mean costs, along with a case load (N0) and an RSV-positivity rate of 27.9% based on a previous RSV study at the KCH and TUTH [5]. This analysis determined that we had to enrol 465 ARI outpatients and 327 ARI inpatients to obtain the optimal sample size of 93 RSV-positive outpatients and 91 RSV-positive inpatients. The enrolment numbers varied from 128 to 900 ARI outpatients and 122 to 910 ARI inpatients, depending on the observed prevalence during the RSV season.

### Study timeline

The study took place during one local respiratory season, from July 2023 until November 2023. RSV testing was, however, continued in children <2 years old admitted with (S)ARI diagnosis until the end of the RSV season in the second week of February 2024 to determine if the characteristics of children enrolled in the cost of illness evaluation differed from children with RSV disease towards the end of the RSV season.

### Data collection

Trained study staff administered questionnaires designed for the RSV GOLD III – Health Economics Study. In contrast to other study sites such as Mozambique [13], where data was collected on paper, data in Nepal were directly recorded electronically using the Electronic Data Capture System Castor [22] and the Kobo Toolbox [23]. Follow-up occurred four to six weeks after discharge. Child and family demographic and clinical data gathering took place *via* direct interviews and hospital records for outpatient and inpatient cases (Figure S1 in the **Online Supplementary Document**).

We used billing data, including out-of-pocket and health system expenditures, to identify resource utilisation, as well as direct medical and overhead costs associated with care received within the facility. We estimated costs from the health system and household perspectives to calculate costs from the societal perspective. The health system perspective covered direct medical costs, with costs calculated using unit prices per service at the KCH and TUTH. We also included patients receiving support for treatment costs from health insurance or social funding sources (not the healthcare provider). Household costs included out-of-pocket medical expenses, non-medical costs, as well as indirect costs from caregivers’ lost income and leisure time (self-reported), with all costs related to acute respiratory infection. We excluded unrelated medication and services from household costs, but included them in societal costs.

### Sample collection

RSV testing occurred as part of the study protocol and was not routine practice at the participating hospitals. Samples were collected as previously described in the ICU Network Study, published elsewhere [5]. While the standard method for RSV testing is typically polymerase chain reaction (PCR), we used the ID Now molecular test device, which has been validated in previous studies, and has shown reliable results when compared to the gold standard for RSV testing (*i.e. *the PCR method), particularly in the Nepali setting [15].

Due to the unexpectedly high RSV positivity rate among the included patients during the study period, all patients <2 years old admitted to the participating hospitals were tested for RSV at two intervals during the study period. Testing all children in this age range made verifying whether the high positivity rates were consistent across the broader population or confined to those with (S)ARI possible.

### Data analysis

We used a bottom-up costing approach to estimate treatment costs for respiratory illness by identifying the resources consumed at the individual patient level, multiplying the quantity of each resource by its respective unit costs, and stratifying the costs by severity of RSV illness, including both medical and non-medical expenses. We estimated caregiver transportation costs based on travel distance and fuel consumption, and calculated indirect costs (including lost income and leisure) by multiplying the hourly income by working hours lost due to caregiving. We imputed missing caregiver wages using the mean average wage of other respondents. We monetised the value of lost leisure time by using the minimum wage in Nepal [16].

Overhead costs were allocated to individual patients based on their length of stay, deducting any registration fee and bed charges already paid. If the calculated administration fee (which included overhead costs, facility expenses, and staff costs) was greater than or equal to the registration fee and bed charges already included, the administration fee was not changed. If the administration fee was lower than the registration fee and bed charges paid, we added the difference to the total administration fee.

Total costs were the sum of direct medical costs, direct non-medical costs, and indirect costs for RSV-positive and non-RSV (S)ARI patients. Since cost data were predominantly non-parametric, we summarised cost data using both median and mean values. Cost data in our study are strongly right-skewed due to a few statistical outliers. We verified the values of these outliers for accuracy and included them in the final analysis, as they accurately reflected the true nature of the distribution of cost data. Costs were collected in NPR and converted to 2023 USD using the annual average exchange rate for 2023 (NPR 132.12 per USD 1). We conducted all statistical analyses separately for RSV-positive and non-RSV (S)ARI patients using Stata BE, version 18.0 (StatCorp LLC, College Station, Texas, USA).

## RESULTS

### Patient characteristics

**Figure 1:**
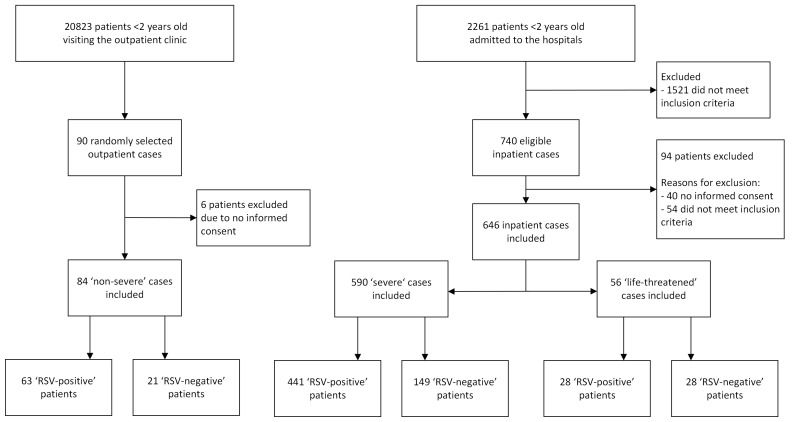
Flow diagram, showing the total numbers of outpatients and inpatients included and excluded from the study. RSV – respiratory syncytial virus.

Between 1 July 2023 and 30 November 2023, 730 patients were enrolled in the study (**Figure 1**), of whom 532 (72.8%) tested positive for RSV: 63 (11.8%) at the outpatient clinic, 441 (82.9%) at the wards (n = 278) and HDU (n = 163), and 28 (5.3%) at the ICU. The mean age of RSV-positive children was 9.1 months for non-severe, 6.7 months for severe, and 4.0 months for life-threatened patients at the time of enrolment (**Table 1**). Most RSV-positive children in the study were male (n = 320, 60.2%). Inpatients accounted for 88.5% of all patients, primarily due to the high burden at the outpatient clinics and the consequent limitation on the number of patients that could be included there. We observed an RSV positivity rate of 72.6% for inpatients with SARI (Figure S2 in the **Online Supplementary Document**). Verification by testing all children <2 years old admitted to the hospitals confirmed the high observed RSV positivity rate (Table S1 in the **Online Supplementary Document**). The median duration of hospitalisation for RSV-positive severe patients was 4 days (interquartile range (IQR) = 3–5), compared to 7 days (IQR = 4.5–10) for life-threatened patients. Twelve children died during the study period, with eight deaths occurring in the hospital. Of these, one-eighth tested positive for RSV. Four more deaths were identified during follow-up, with one testing positive for RSV (Tables S2 and S3 in the **Online Supplementary Document**).

**Table 1:** Demographics and clinical characteristics of RSV-positive and non-RSV (S)ARI patients <2 years old, by severity level*

	RSV positive				Non-RSV (S)ARI				All patients
	**Non-severe**	**Severe**	**Life-threatened**	**Total**	**Non-severe**	**Severe**	**Life-threatened**	**Total**	**Overall**
**Total number of participants**	63	441	28	532	21	149	28	198	730
**Age in months, x̄ (SD)**	9.1 (5.2)	6.7 (5.6)	4.0 (5.0)	6.9 (5.6)	8.2 (5.3)	7.2 (6.5)	3.1 (4.5)	6.7 (6.3)	6.8 (5.8)
**Female**	25 (39.7)	178 (40.4)	9 (32.1)	212 (39.9)	7 (33.3)	44 (29.5)	10 (35.7)	61 (30.8)	273 (37.4)
**Household size, MD (IQR)**	4 (3–5)	4 (4–6)	5 (4–6)	4 (4–6)	4 (3–5)	4 (4–6)	4 (4–5)	4 (4–5)	4 (4–5)
**Level of education primary caregiver secondary school or higher**	42 (66.7)	284 (64.4)	16 (57.1)	342 (64.3)	17 (81.0)	91 (61.1)	20 (71.4)	128 (64.7)	470 (64.4)
**Distance to healthcare facility in km, x̄ (SD)**	3 (4.8)	15 (3.4)	0 (0.0)	18 (3.4)	0 (0.0)	8 (5.4)	2 (7.1)	10 (5.1)	28 (3.8)
**Physician's diagnosis**									
Bronchiolitis	25 (39.7)	227 (51.5)	10 (35.7)	262 (49.3)	10 (47.6)	48 (32.2)	5 (17.9)	63 (31.8)	325 (44.5)
Pneumonia	6 (9.5)	170 (38.6)	17 (60.7)	193 (36.3)	4 (19.1)	76 (51.0)	22 (78.6)	102 (51.5)	295 (40.4)
URTI	2 (3.7)	0 (0.0)	0 (0.0)	2 (0.34)	3 (14.3)	0 (0.0)	0 (0.0)	3 (1.5)	5 (0.78)
Other LRTI	27 (42.9)	11 (2.5)	0 (0.0)	38 (7.1)	4 (19.1)	5 (3.4)	0 (0.0)	9 (4.6)	47 (6.4)
Other	3 (4.8)	23 (8.3)	11 (5.8)	37 (7.0)	0 (0.0)	20 (13.4)	1 (3.6)	21 (10.6)	58 (8.0)
**Prematurity**	0 (0.0)	27 (6.1)	2 (7.1)	29 (5.5)	0 (0.0)	12 (8.1)	5 (18.5)	17 (8.6)	46 (6.3)
**Comorbidity**	0 (0.0)	17 (3.9)	7 (25.0)	24 (4.5)	1 (4.8)	19 (12.8)	11 (39.3)	31 (15.7)	55 (7.5)
**Length of stay in days, MD (IQR)**	N/A	4 (3–5)	7 (4.5–10)	4 (3–6)	N/A	4 (3–6)	8 (5–12.5)	4 (3–7)	4 (3–6)
**Previous medical consultation**	21 (33.3)	214 (48.6)	14 (51.9)	249 (47.0)	6 (28.6)	65 (43.6)	15 (55.6)	86 (43.7)	335 (46.1)
**Follow-up care**	17 (27.0)	183 (42.2)	5 (21.7)	205 (39.4)	3 (14.3)	70 (47.3)	7 (31.8)	80 (41.9)	285 (40.1)
**Mortality**	–	–	2 (7.1)	2 (0.4)	–	–	10 (35.7)	10 (5.1)	12 (1.6)

IQR – interquartile range, MD – median, RSV – respiratory syncytial virus, (S)ARI – (severe) acute respiratory illness, LRTI – lower respiratory tract infection, URTI – upper respiratory tract infection, x̄ – mean

*Values presented as n (%) unless specified otherwise.

### Cost per episode

#### Societal costs

The mean total societal costs per RSV episode were USD 43 (95% CI = 25–62) for non-severe patients, USD 312 (95% CI = 293–332) for severe patients, and USD 664 (95% CI = 381–947) for life-threatened patients. About half of the total costs were direct medical costs. Overall, costs for non-RSV (S)ARI patients were higher, with life-threatened patients showing the largest difference (USD 810 vs USD 664; *P* = 0.01) (**Table 2**). When comparing outpatients and inpatients, the treatment costs for RSV patients were slightly lower than those for non-RSV (S)ARI (Table S4 in the **Online Supplementary Document**). In particular, medical costs for non-RSV life-threatening cases were higher (Table S5 in the **Online Supplementary Document**).

**Table 2:** Summary of societal cost (in 2023 USD) per lower respiratory tract infection episode for RSV-positive and non-RSV (severe) acute respiratory illness patients <2 years old, by severity level

	RSV-positive (n = 532)			Non-RSV (S)ARI (n = 198)		
	**Non-severe (n = 63)**	**Severe (n = 441)**	**Life-threatened (n = 28)**	**Non-severe (n = 21)**	**Severe (n = 149)**	**Life-threatened (n = 28)**
**Total costs**						
x̄ (95% CI)	43.43 (24.77–62.09)	312.33 (292.81–331.85)	664.06 (381.39–946.73)	70.09 (20.68–119.51)	364.41 (317.25–411.57)	809.92 (612.70–1,005.15)
MD (IQR)	28.30 (24.68–39.46)	260.90 (176.88–386.43)	486.82 (319.57–649.07)	31.00 (24.88–47.82)	295.99 (194.47–402.10)	629.88 (434.61–1,152.83)
**Direct medical costs**						
x̄ (95% CI)	21.61 (20.29–22.94)	125.78 (118.28–133.28)	305.63 (204.68–406.57)	24.66 (20.11–29.22)	145.59 (127.67–163.52)	471.27 (322.86–619.68)
MD (IQR)	20.43 (18.92–23.99)	107.07 (81.95–143.98)	236.27 (154.16–321.61)	19.04 (18.03–26.11)	113.70 (87.51–153.86)	277.93 (232.14–566.14)
Hospitality/facility-based fees						
*x̄ (95% CI)*	16.14 (15.53–16.75)	74.57 (70.07–79.07)	138.68 (104.61–172.75)	17.19 (15.97–18.42)	84.65 (74.12–95.18)	149.47 (108.10–190.84)
*MD (IQR)*	15.09 (15.09–18.59)	60.72 (45.63–81.44)	106.35 (75.80–155.74)	15.46 (15.09–18.59)	61.47 (47.76–90.89)	136.15 (90.51–182.75)
Medication costs						
*x̄ (95% CI)*	2.57 (2.16–2.98)	13.67 (12.59–14.74)	35.52 (22.82–48.22)	2.89 (1.61–4.17)	16.16 (13.53–18.79)	55.64 (35.01–76.27)
*MD (IQR)*	2.45 (1.20–3.65)	11.01 (7.38–16.55)	19.53 (13.86–42.97)	2.14 (0.90–3.38)	11.14 (7.98–17.58)	35.37 (16.45–81.37)
Laboratory costs						
*x̄ (95% CI)*	1.00 (0.22–1.77)	12.61 (11.60–13.61)	33.99 (20.37–47.61)	3.04 (0.00–6.34)	17.38 (14.28–20.49)	73.38 (34.21–112.55)
*MD (IQR)*	0.00 (0.00–0.00)	9.96 (7.52–14.92)	21.81 (12.22–48.13)	0.00 (0.00–4.89)	11.09 (7.52–19.06)	33.79 (21.43–55.84)
Imaging costs						
*x̄ (95% CI)*	2.58 (2.16–3.01)	4.23 (3.85–4.61)	11.57 (0.90–22.23)	2.61 (2.09–3.13)	6.98 (4.41–9.55)	21.58 (11.20–31.96)
*MD (IQR)*	2.26 (2.26–3.16)	2.26 (2.26–4.51)	4.16 (2.26–8.50)	2.26 (2.26–3.16)	2.26 (2.26–6.77)	9.02 (4.51–30.53)
Procedure costs						
*x̄ (95% CI)*	0.16 (0.04–0.27)	7.19 (5.80–8.58)	58.24 (23.52–92.95)	0.86 (0.00–2.11)	5.52 (3.82–7.22)	84.13 (37.98–130.28)
*MD (IQR)*	0.00 (0.00–0.00)	3.76 (1.88–7.37)	27.26 (11.88–49.26)	0.00 (0.00–0.90)	2.19 (0.71–5.68)	24.82 (13.91–103.78)
Miscellaneous costs						
*x̄ (95% CI)*	0.46 (0.27–0.66)	14.68 (13.67–15.68)	37.93 (31.08–44.78)	3.31 (0.96–5.65)	16.38 (14.25–18.51)	82.41 (57.20–107.62)
*MD (IQR)*	0.19 (0.00–0.86)	11.78 (7.93–18.12)	37.26 (26.52–47.63)	1.03 (0.15–7.34)	12.42 (8.51–19.15)	58.28 (30.82–111.59)
Direct non-medical costs*						
x̄ (95% CI)	8.32 (3.00–13.65)	63.85 (54.51–73.20)	118.92 (69.17–168.67)	34.98 (0.00–81.46)	65.23 (52.58–77.88)	153.74 (113.86–193.62)
MD (IQR)	1.80 (0.84–5.83)	36.10 (19.93–65.65)	79.49 (55.46–138.11)	7.52 (0.90–13.99)	39.14 (20.31–76.71)	137.47 (67.83–193.35)
Transport costs						
*x̄ (95% CI)*	6.37 (1.77–10.96)	37.22 (28.20–46.23)	46.33 (25.56–67.11)	32.69 (0.00–78.92)	32.09 (21.28–42.91)	79.83 (49.57–110.09)
*MD (IQR)*	1.35 (0.71–4.70)	8.27 (1.62–24.07)	19.89 (9.40–71.07)	5.26 (0.75–12.03)	8.80 (1.48–24.59)	52.04 (14.66–142.89)
Meal costs						
*x̄ (95% CI)*	1.92 (0.00–4.10)	26.33 (24.10–28.56)	51.33 (35.50–67.16)	2.25 (0.21–4.28)	32.05 (26.38–37.71)	72.39 (45.03–99.76)
*MD (IQR)*	0.00 (0.00–0.90)	21.66 (11.28–34.59)	45.91 (24.82–57.08)	0.00 (0.00–2.26)	24.82 (13.24–37.90)	45.72 (18.80–117.09)
**Indirect costs**						
*x̄ (95% CI)*	13.50 (0.00–27.20)	124.69 (112.75–136.75)	259.70 (106.02–413.39)	10.46 (2.24–18.67)	153.59 (122.18–185.00)	201.37 (131.96–270.78)
*MD (IQR)*	3.40 (0.63–9.61)	90.50 (48.41–157.27)	156.71 (99.27–265.39)	2.27 (1.51–7.63)	106.13 (48.41–187.63)	143.86 (42.36–295.54)
Lost income						
*x̄ (95% CI)*	1.66 (0.00–4.08)	11.16 (8.70–13.61)	17.47 (5.91–29.03)	2.67 (0.14–5.20)	19.54 (14.13–24.95)	21.51 (6.34–36.69)
*MD (IQR)*	0.00 (0.00–0.00)	0.00 (0.00–0.00)	0.00 (0.00–35.09)	0.00 (0.00–0.00)	0.00 (0.00–35.09)	0.00 (0.00–37.60)
Lost productivity						
*x̄ (95% CI)*	8.73 (0.00–18.94)	54.67 (45.52–63.82)	141.47 (10.15–272.79)	4.30 (0.30–8.30)	71.17 (44.57–97.76)	72.97 (39.57–106.36)
*MD (IQR)*	0.00 (0.00–6.65)	20.99 (0.00–66.37)	55.97 (4.37–96.19)	0.00 (0.00–3.85)	35.16 (0.00–78.70)	55.97 (5.25–100.56)
Lost leisure						
*x̄ (95% CI)*	3.11 (1.86–4.37)	58.86 (55.05–62.67)	100.76 (73.36–128.17)	3.49 (0.67–6.31)	62.88 (55.20–70.56)	106.89 (62.08–151.69)
*MD (IQR)*	2.27 (0.25–4.16)	60.51 (36.31–72.62)	96.82 (60.51–128.97)	1.51 (0.00–3.40)	60.51 (30.64–72.62)	66.57 (18.15–136.16)

CI – confidence interval, IQR – interquartile range, MD – median, RSV – respiratory syncytial virus, (S)ARI – (severe) acute respiratory illness, x̄ – mean

*Besides transport and meals, caretaker costs and lodging costs are included here; however, only few cases have costs here and are therefore not shown in the table.

#### Health system costs

Health system costs (**Table 3**) were comparable for severe and life-threatened RSV patients, with USD 58 (95% CI = 54–62) and USD 57 (95% CI = 32–83) per illness episode. Main cost drivers were administrative fees, including registration fees, bed charges, and staff costs. Similar results were found for non-RSV (S)ARI patients, with an additional average of USD 10.

**Table 3:** Direct medical cost (in 2023 USD) covered by the health system per lower respiratory tract infection episode for RSV-positive and non-RSV (severe) acute respiratory illness patients <2 years old, by severity level

	RSV-positive (n = 532)			Non-RSV (S)ARI (n = 198)		
	**Non-severe (n = 63)**	**Severe (n = 441)**	**Life-threatened (n = 28)**	**Non-severe (n = 21)**	**Severe (n = 149)**	**Life-threatened (n = 28)**
Direct medical costs*						
x̄ (95% CI)	15.78 (15.04–16.52)	58.09 (53.87–62.31)	57.38 (31.86–82.90)	15.93 (15.26–16.60)	67.89 (59.05–76.73)	68.04 (40.47–95.60)
MD (IQR)	14.71 (14.71–17.84)	49.11 (39.39–65.70)	40.41 (13.66–67.33)	14.71 (14.71–17.84)	58.09 (42.29–74.30)	52.44 (5.40–89.89)
Hospitality/facility-based fees						
*x̄ (95% CI)*	15.40 (14.80–16.00)	55.23 (51.67–58.79)	52.63 (34.19–71.07)	15.93 (15.26–16.60)	65.84 (57.39–74.30)	56.72 (37.25–76.19)
*MD (IQR)*	14.71 (14.71–17.84)	46.75 (38.49–61.65)	40.36 (21.94–67.33)	14.71 (14.71–17.84)	58.09 (41.54–73.17)	45.41 (5.40–80.87)
Medication costs						
*x̄ (95% CI)*	0.13 (0.00–0.27)	1.02 (0.46–1.57)	1.19 (0.11–2.26)	0.00 (0.00–0.00)	0.63 (0.22–1.05)	3.04 (0.00–8.44)
*MD (IQR)*	0.00 (0.00–0.00)	0.00 (0.00–0.00)	0.00 (0.00–0.00)	0.00 (0.00–0.00)	0.00 (0.00–0.00)	0.00 (0.00–0.00)
Laboratory costs						
*x̄ (95% CI)*	0.23 (0.00–0.69)	0.68 (0.24–1.12)	0.00 (0.00–0.00)	0.00 (0.00–0.00)	0.82 (0.04–1.60)	0.62 (0.00–1.62)
*MD (IQR)*	0.00 (0.00–0.00)	0.00 (0.00–0.00)	0.00 (0.00–0.00)	0.00 (0.00–0.00)	0.00 (0.00–0.00)	0.00 (0.00–0.00)
Imaging costs						
*x̄ (95% CI)*	0.18 (0.00–0.38)	0.15 (0.06–0.24)	0.00 (0.00–0.00)	0.00 (0.00–0.00)	0.33 (0.00–0.66)	0.72 (0.00–2.06)
*MD (IQR)*	0.00 (0.00–0.00)	0.00 (0.00–0.00)	0.00 (0.00–0.00)	0.00 (0.00–0.00)	0.00 (0.00–0.00)	0.00 (0.00–0.00)
Procedure costs						
*x̄ (95% CI)*	0.00 (0.00–0.00)	1.06 (0.41–1.71)	5.77 (0.00–14.71)	0.00 (0.00–0.00)	0.27 (0.05–0.48)	6.66 (0.00–13.71)
*MD (IQR)*	0.00 (0.00–0.00)	0.00 (0.00–0.00)	0.00 (0.00–0.00)	0.00 (0.00–0.00)	0.00 (0.00–0.00)	0.00 (0.00–0.00)

CI – confidence interval, IQR – interquartile range, MD – median, RSV – respiratory syncytial virus, (S)ARI – (severe) acute respiratory illness, x̄ – mean

*Miscellaneous costs are part of direct medical costs; however, those costs are >95% covered by the households and are therefore not shown in the table.

#### Household costs

Overall, the National Health Insurance System covered 28 patients (3.8%) included in the study. Of the 532 RSV-positive patients, all caregivers reported out-of-pocket household costs (**Table 4**). The mean total out-of-pocket household costs were USD 19 (95% CI = 10–28), USD 200 (95% CI = 187–214), and USD 472 (95% CI = 324–620) for non-severe, severe, and life-threatened patients, respectively. Direct medical expenses were the largest component of household costs for all caregivers, contributing 30.8%–52.6% to the overall costs paid by households. Direct non-medical costs such as transport and food were reported by 525 (98.1%) of the caregivers, differing between USD 8 and USD 119. For severe and life-threatened RSV-positive inpatients, indirect costs were between 58.9% and 175.4% of the mean monthly household income in Nepal.

**Table 4:** Household costs (in 2023 USD) per lower respiratory tract infection episode for RSV-positive and non-RSV (severe) acute respiratory illness patients <2 years old, by severity level

	RSV-positive (n = 532)			Non-RSV (S)ARI (n = 198)		
	**Non-severe (n = 63)**	**Severe (n = 441)**	**Life-threatened (n = 28)**	**Non-severe (n = 21)**	**Severe (n = 149)**	**Life-threatened (n = 28)**
**Total costs**						
x̄ (95% CI)	18.92 (9.92–27.93)	200.48 (186.54–214.43)	472.31 (324.44–620.19)	49.86 (1.01–98.72)	225.35 (197.93–252.77)	670.44 (485.34–855.55)
MD (IQR)	11.00 (7.36–16.79)	165.49 (109.23–246.14)	362.36 (265.49–503.16)	13.15 (9.88–33.11)	184.89 (119.50–270.86)	515.62 (314.32–842.39)
**Direct medical costs**						
x̄ (95% CI)	5.83 (4.61–7.05)	67.70 (62.35–73.04)	248.25 (168.00–328.49)	8.73 (4.45–13.02)	77.70 (66.18–89.22)	403.23 (266.92–539.54)
MD (IQR)	5.23 (3.03–7.45)	52.27 (33.86–85.41)	189.19 (124.55–321.61)	4.14 (2.63–8.27)	55.62 (37.14–88.67)	233.66 (166.23–534.39)
Hospitality/facility based fees						
*x̄ (95% CI)*	0.74 (0.49–0.99)	19.34 (16.78–21.90)	86.05 (63.92–108.18)	1.27 (0.54–1.99)	18.81 (14.49–23.12)	92.75 (60.16–125.34)
*MD (IQR)*	0.38 (0.38–0.75)	9.02 (2.26–25.95)	65.24 (45.88–103.03)	0.38 (0.38–1.13)	4.89 (2.26–27.07)	68.81 (53.40–92.13)
Medication costs						
*x̄ (95% CI)*	2.44 (2.04–2.83)	12.65 (11.66–13.64)	34.33 (21.93–46.74)	2.89 (1.61–4.17)	15.53 (12.93–18.13)	52.60 (32.95–72.25)
*MD (IQR)*	2.41 (1.17–3.37)	10.50 (6.84–15.67)	19.53 (13.86–42.97)	2.14 (0.90–3.38)	10.77 (7.75 –17.28)	34.61 (16.45–76.63)
Laboratory costs						
*x̄ (95% CI)*	0.76 (0.12–1.40)	11.93 (10.97–12.88)	33.99 (20.37–47.61)	3.04 (0.00–6.34)	16.57 (13.48–19.66)	72.75 (33.88–111.63)
*MD (IQR)*	0.00 (0.00–0.00)	9.78 (7.52 –13.72)	21.81 (12.22–48.13)	0.00 (0.00–0.00)	10.30 (7.52–17.49)	33.79 (21.43–55.84)
Imaging costs						
*x̄ (95% CI)*	2.41 (1.96–2.85)	4.08 (3.70–4.47)	11.57 (0.90–22.23)	2.61 (2.09–3.13)	6.65 (4.16–9.14)	20.86 (10.58–31.14)
*MD (IQR)*	2.26 (2.26–2.26)	2.26 (2.26–4.51)	2.26 (2.26–8.50)	2.26 (2.26–3.16)	2.26 (2.26–6.77)	9.02 (4.51–27.30)
Procedure costs						
*x̄ (95% CI)*	0.16 (0.04–0.27)	6.14 (4.84–7.44)	52.46 (25.32–79.61)	0.86 (0.00–2.11)	5.30 (3.59–7.01)	77.47 (34.52–120.41)
*MD (IQR)*	0.00 (0.00–0.00)	2.73 (1.50–5.81)	27.07 (11.88–49.26)	0.00 (0.00–0.00)	1.88 (0.66–5.64)	24.82 (13.91–90.25)
Miscellaneous costs						
*x̄ (95% CI)*	0.46 (0.27–0.66)	14.56 (13.55–15.57)	37.93 (31.08–44.78)	3.31 (0.96–5.65)	16.30 (14.19–18.42)	81.89 (56.97–106.82)
*MD (IQR)*	0.19 (0.00–0.86)	11.72 (7.86–18.04)	37.26 (26.52–47.63)	1.03 (0.15–7.34)	12.42 (8.44–19.15)	58.28 (30.82–111.59)
Direct non-medical costs*						
x̄ (95% CI)	8.32 (2.99–13.65)	63.85 (54.51–73.20)	118.92 (69.17–168.67)	34.98 (0.00–81.46)	65.23 (52.58–77.88)	153.74 (113.86–193.62)
MD (IQR)	1.80 (0.84–5.83)	36.10 (19.93–65.65)	79.49 (55.46–138.11)	7.52 (0.90–13.99)	39.14 (20.31–76.71)	137.47 (67.83–193.35)
Transport costs						
*x̄ (95% CI)*	6.37 (1.77–10.96)	37.22 (28.20–46.23)	46.33 (25.56–67.11)	32.69 (0.00–78.92)	32.09 (21.28–42.91)	79.83 (49.57–110.09)
*MD (IQR)*	1.35 (0.71–4.70)	8.27 (1.62–24.07)	19.89 (9.40–71.07)	5.26 (0.75–12.03)	8.80 (1.48–24.59)	52.04 (14.66–142.89)
Meal costs						
*x̄ (95% CI)*	1.92 (0.00–4.10)	26.33 (24.10–28.56)	51.33 (35.50–67.16)	2.25 (0.21–4.28)	32.05 (26.38–37.71)	72.39 (45.03–99.76)
*MD (IQR)*	0.00 (0.00–0.00)	21.66 (11.28–34.59)	45.91 (24.82–57.08)	0.00 (0.00–0.00)	24.82 (13.24–37.90)	45.72 (18.80–117.09)
**Indirect costs**						
x̄ (95% CI)	4.77 (1.23–8.32)	70.02 (64.85–75.19)	118.24 (86.22–150.25)	6.16 (1.50–10.81)	82.42 (71.32–93.53)	128.40 (82.00–174.81)
MD (IQR)	2.27 (0.44- 4.54)	60.51 (36.50–86.84)	96.82 (60.51–159.06)	2.27 (1.51–4.54)	62.40 (36.56–104.52)	87.90 (36.31–222.08)
Lost income						
*x̄ (95% CI)*	1.66 (0.00–4.08)	11.16 (8.70–13.61)	17.47 (5.91–29.03)	2.67 (0.14–5.20)	19.54 (14.13–24.95)	21.51 (6.34–36.69)
*MD (IQR)*	0.00 (0.00–0.00)	0.00 (0.00–0.00)	0.00 (0.00–35.09)	0.00 (0.00–0.00)	0.00 (0.00–35.09)	0.00 (0.00–37.60)
Lost leisure						
*x̄ (95% CI)*	3.11 (1.86–4.37)	58.66 (55.05–62.67)	100.76 (73.36–128.17)	3.49 (0.67–6.31)	62.88 (55.20–70.56)	106.89 (62.08–151.70)
*MD (IQR)*	2.27 (0.25–4.16)	60.51 (36.31–72.62)	96.82 (60.51–128.97)	1.51 (0.00–3.40)	60.51 (30.64–72.62)	66.57 (18.15–136.16)

CI – confidence interval, IQR – interquartile range, MD – median, RSV – respiratory syncytial virus, (S)ARI – (severe) acute respiratory illness, x̄ – mean

*Besides transport and meal costs, caretaker and lodging costs are included here; however, only a few cases have costs and are therefore not shown in this table.

## DISCUSSION

Our findings show substantial direct medical, non-medical, and indirect costs of (S)ARI-related illness in children <2 years old visiting two public hospitals in Kathmandu, Nepal. The medical costs of RSV-related hospital admissions have a substantial economic impact not only on the health system, but also on households in Nepal.

Existing literature highlights significant variability in the total costs associated with RSV-related hospital admissions across different countries and regions [17]. In our study, the total inpatient costs for RSV-positive children were USD 312 for severe cases and USD 664 for life-threatened cases, which are much higher compared to other Asian LMICs such as Bangladesh (USD 94 in 2010; USD 131 when adjusted for inflation to 2023) and Vietnam (USD 165 in 2022; USD 172 when adjusted for inflation to 2023). Outpatient costs for RSV-positive cases in our study were USD 43, and while these findings are comparable to data from Vietnam (USD 52; USD 54 when adjusted for inflation to 2023), the costs for hospitalised RSV patients in Nepal were significantly higher across all cost categories. Higher cost per RSV illness episode observed in our study could potentially be linked to several factors, including the following: the availability of ICUs at the study hospitals, which tend to be more expensive; a high proportion of enrolled children being admitted to HDU; and the completeness and quality of data collection. An unpublished study on the costs of pneumonia treatment in children 0–59 months of age conducted across five hospitals in Nepal reported an average cost of USD 177 in 2016 (USD 225 when adjusted for inflation to 2023) per pneumonia case [18]. This study suggests that, despite potential regional cost variations, our estimate for the cost of RSV illness adds a valuable perspective on the economic burden of RSV disease in Nepal.

Differences in indirect costs, mostly driven by lost income, are also much higher in our study (USD 125 for severe cases and USD 260 for life-threatened cases) when compared to Bangladesh (USD 19; USD 27 when adjusted for inflation to 2023) and Vietnam (USD 19; USD 20 when adjusted for inflation to 2023). These differences could reflect varying salary levels across countries but could also be influenced by the presence of different caregivers accompanying patients to the hospital. For instance, in Nepal, fathers, who are typically the primary income earners, often also accompany patients; whereas elsewhere, mothers, who may not be working, more commonly serve as the primary caregiver. These findings underscore the broader economic impact of RSV, extending beyond direct medical expenses. They also highlight the need for a comprehensive country-specific evaluation that encompasses the direct and indirect burden of RSV to inform health policy and effective resource allocation.

Household out-of-pocket costs for inpatients in our study were substantial, placing a significant financial burden on families. The mean direct medical costs for caregivers reporting inpatient care costs accounted for 73.9% of Nepal’s all-source health expenditure per capita, which was USD 65 in 2021 (USD 73 when adjusted for inflation to 2023 USD) [19]. This finding highlights the significant burden that RSV imposes on households. Previous research has defined catastrophic health expenditure as healthcare costs exceeding 10% of a household’s annual income [20]. Keeping this in mind, if caregivers reporting income loss were the only income earners in their household, the mean direct medical and non-medical costs per RSV illness episode for those requiring inpatient care would likely qualify as catastrophic. Although we did not measure household income in this study, projecting the annual cost of RSV illnesses in Nepal using an ARI incidence rate of approximately 2.1% [3] can provide insight into the national household economic burden and the potential risk of impoverishment due to RSV illnesses. Reducing the burden of RSV, and thereby reducing the financial strain on households, is crucial for advancing efforts to lower childhood mortality and combat poverty.

Medical costs are a substantial part of our study and are slightly higher in the non-RSV (S)ARI group compared to the RSV patients. The largest difference is for medication and laboratory costs. This difference could be because healthcare providers have access to the point-of-care test results, leading to changes in their prescribing behaviour (*e.g.* reducing antibiotic prescriptions and not testing for other pathogens). This finding indicates the potential of point-of-care testing in influencing clinical decision-making and improving patient management by minimising unnecessary antibiotic use and testing procedures, thereby lowering medical and laboratory costs.

The overall cost in 2023 accounted for 3.3% of GDP per capita for non-severe cases, 23.6% for severe cases, and 50.2% for life-threatened cases of RSV infection. The high cost, particularly for severe and life-threatened cases, suggests a significant financial burden on both households and the government. For low-income families, where GDP per capita is already limited, these healthcare costs could exacerbate poverty, pushing households into financial distress or forcing them to forego necessary medical care. Additionally, the high governmental costs associated with treating severe cases could strain public healthcare budgets, potentially limiting resources available for other critical services.

This is the first prospective study to evaluate direct medical, direct non-medical, and indirect costs for households and the health system related to (S)ARI in Nepal. While it provides valuable data for public health decision-making, it also has several limitations. First, the study population consists of patients from two public hospitals in the capital of Nepal, limiting the generalisability of the findings to the whole country. Second, the fact that we include fewer outpatients than required by the sample size calculations may mean that costs incurred by outpatients are not fully representative. Third, the results presented here likely underestimate the total economic burden of RSV illness in Nepal. Our study was limited to inpatients and outpatients at secondary and tertiary public facilities and did not capture costs associated with milder cases treated in an outpatient setting, at smaller health facilities, or at private facilities. Fourth, follow-up conducted via phone calls may have contributed to lower response rates when compared to regular in-person follow-up visits. We note, however, the high study adherence among our participants. Fifth, reliance on self-reported cost data from parents/caregivers may compromise the validity of the findings. Sixth, costs in private health facilities may potentially be higher due to factors such as specialised services, greater use of advanced medical technology, and the inclusion of overhead expenses not typically covered in public facilities [21]. Finally, we did not account for genetic factors that influence susceptibility to RSV, and we only captured incurred costs without considering the quality of care received.

## CONCLUSIONS

Our study provides a detailed description of the cost and economic burden of RSV and non-RSV (S)ARI in children <2 years old in Kathmandu, Nepal. The burden on the health system and on households is substantial. Medical costs associated with severe and life-threatening RSV in urban Nepal are particularly high for its households. Detailed quantification of RSV-related costs is essential for informed decision-making, prioritising future preventive interventions, and achieving effective health resource allocation. Additionally, these data could be valuable for estimating the overall benefit of potential RSV immunisation strategies (*e.g.* with maternal vaccine and/or monoclonal antibody) in Nepal and other settings and will support cost-effectiveness modelling for RSV prevention strategies. Furthermore, future studies should investigate the economic burden in rural areas of Nepal, as well as the impact of (S)ARI illness in the private healthcare sector.

## Additional material


Online Supplementary Document
