# Supplementary material for: Assessing the cost of illness of RSV and non-RSV acute respiratory infections in Nepali children
Source: J Glob Health. 2025 Apr 11;15:04092. doi: 10.7189/jogh.15.04092 (PMC11986276; doi:10.7189/jogh.15.04092)
Supplement: Online Supplementary Document [file jogh-15-04092-s001.pdf]

Supplementary material to:
Rave N, Sharma AK, Chapagain RH, Shrestha R, Nguyen A, Pecenka C, Shaaban FL, Joshi P, Bont LJ; RSV GOLD III – Health Economics Study Group. Assessing the cost of illness of RSV and non-RSV acute respiratory infections in Nepali children. J Glob Health. 2025;15:04092.

With the RSV GOLD III – Health Economics Study we evaluate costs associated with acute lower respiratory tract infections (LRTIs) in children <2 years old in four developing countries (Ghana, Mozambique, Nepal, and Nigeria), during one local respiratory season. Here, we provide supplementary material related to the study conducted in Nepal.

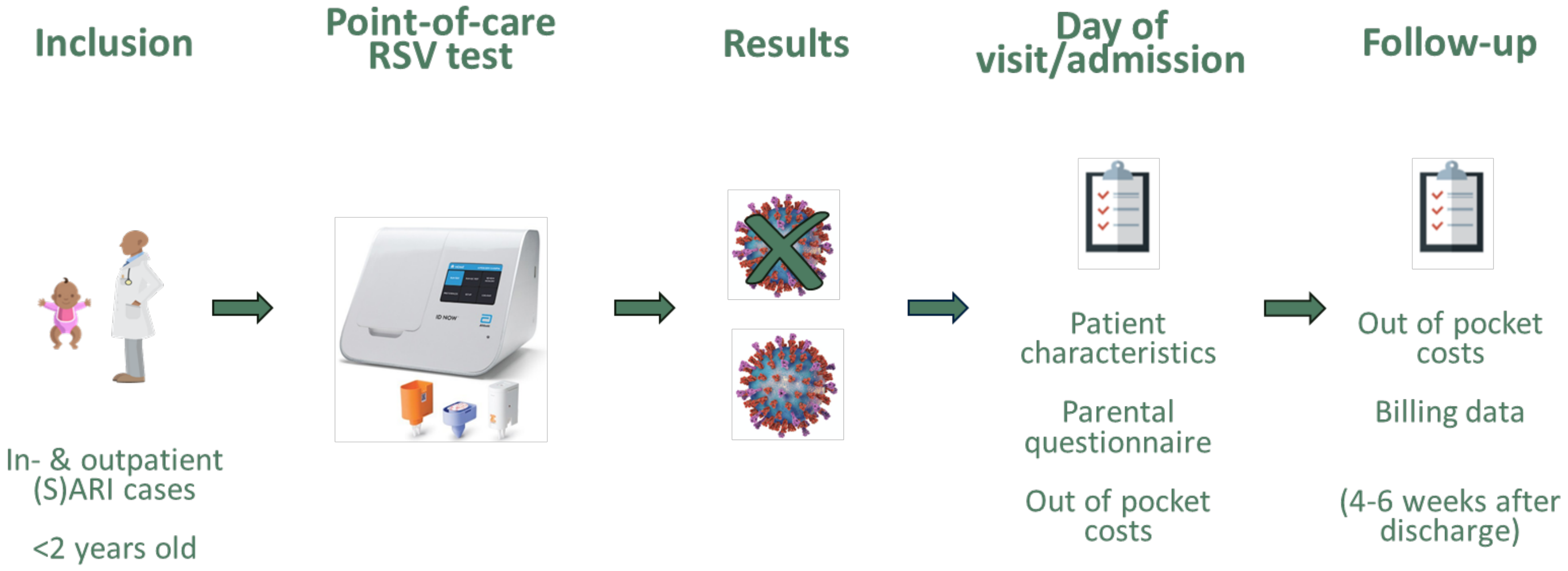

Figure S1. Overview of the clinical process and data collection. RSV – respiratory syncytial virus, (S)ARI – (severe) acute respiratory infection.

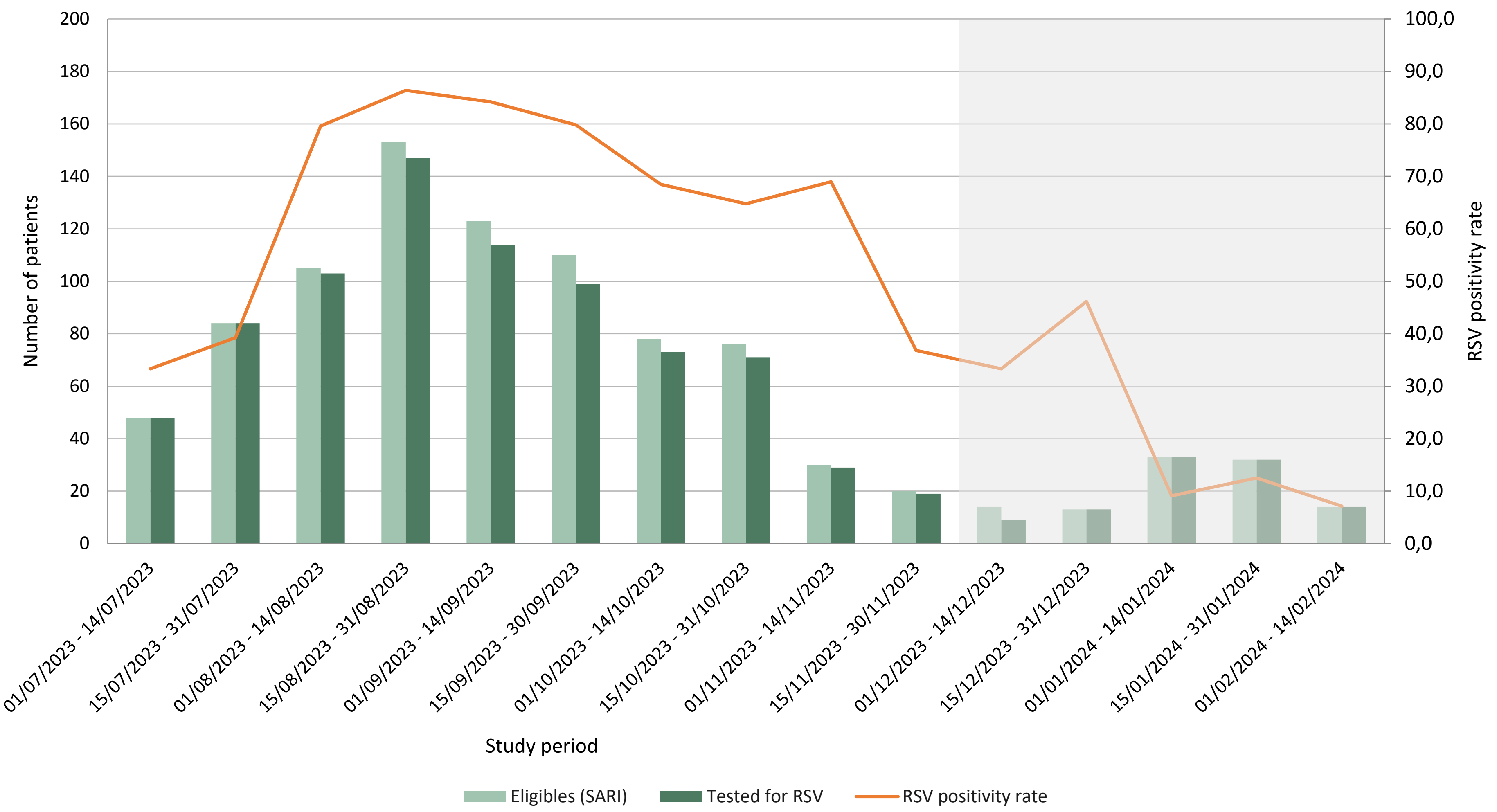

Figure S2. Biweekly numbers of eligible and tested hospitalised children <2 years old, as well as RSV positivity rates, during the original study period and additional testing period. RSV – respiratory syncytial virus, (S)ARI – (severe) acute respiratory infection.

Table S1. RSV testing results for all children <2 years old admitted to the participating hospitals, with testing conducted at two one-week intervals during the study period*

	(S)ARI cases (meet inclusion criteria)		Exclusion (do not meet inclusion criteria)				
	**RSV-positive**	**RSV-negative**	**RSV-positive**	**RSV-negative**	**Total**	**Overall RSV positivity rate**	**Diagnosis and remarks for excluded children who were RSV-positive by testing**
**Week 1**							
15th OCT 2023	7	2	5	9	23	52.2%	Bronchiolitis n=2 (excluded as they spent more than three days in emergency); Down syndrome n=1; Very late onset neonatal sepsis n=1; Suspected meningitis n=1
16th OCT 2023	3	2	3	9	17	35.3%	Sepsis n=1; ACHD n=1; Pneumonia n=1 (symptoms of more than 10 days)
17th OCT 2023	4	1	0	4	9	44.4%	N/A
18th OCT 2023	2	1	2	1	6	66.7%	Pneumonia n=1 (referred case from other hospital with long treatment); Pertussis like illness n=1 (admitted after three days of stay in observation ward)
19th OCT 2023	3	3	3	3	12	50.0%	Seizure Disorder n=1; Pneumonia n=1 (referred case from other hospital after long treatment); Pneumonia n=1 (admitted after more than three days of stay in ward)
**TOTAL**	19	9	13	26	67	47.8%	

Table S1. Continued

**Week 2**							
5th NOV 2023	4	3	3	11	21	33.3%	Pneumonia n=1; Bronchiolitis n=1; Respiratory distress syndrome n=1 (all referred form anther hospital with at least four days of admission)
6th NOV 2023	3	0	1	0	4	100%	N/A
7th NOV 2023	0	1	2	5	8	25.0%	Pneumonia n=1 (admitted to ward after five days of stay in observation ward)
8th NOV 2023	1	0	1	5	7	28.6%	Bronchiolitis n=1 (referred case from other hospital); Pertussis like illness n=1 (admitted after stay in observation ward for four days)
9th NOV 2023	1	1	0	0	2	50.0%	N/A
**TOTAL**	9	6	8	23	46	37.0%	

ACHD – acyanotic congenital heart disease, (S)ARI – (severe) acute respiratory illness, RSV – respiratory syncytial virus.
*Values presented as n.

Table S2. Demographics and clinical characteristics of RSV-related deaths compared to non-RSV (severe) acute respiratory illness mortality cases*

	Fatal RSV infection	Fatal (S)ARI
**Total number of participants**	2	10
**Age in months, X̄ (SD)**	4.0 (4.2)	3.2 (2.8)
**Female**	0 (0.0)	2 (20.0)
**Median household size, MD (IQR)**	4.5 (4–5)	5.0 (4–5)
**Prematurity**	1 (50.0)	1 (10.0)
**Comorbidity**	1 (50.0)	5 (50.0)
**Fully vaccinated according to local immunisation schedule and age**	1 (50.0)	9 (90.0)
**Maternal immunisation**	2 (100.0)	10 (100.0)
**Presentation upon admission**		
Time between onset of symptoms and hospital admission in days, X̄ (SD)	2.0 (1.4)	3.1 (2.0)
Fever (temperature >38ºC)	1 (50.0)	5 (50.0)
Oxygen saturation on room air, X̄ (SD)	86.5 (0.71)	80.0 (6.36)
Concomitant non-respiratory diagnosis	0 (0.0)	0 (0.0)
**Disease management**		
Oxygen support	2 (100.0)	6 (60.0)
Non-invasive ventilatory support (CPAP or HFNC)	1 (50.0)	2 (20.0)
Mechanical ventilation	0 (0.0)	5 (50.0)

Table S2. Continued

Systemic corticosteroids administered	0 (0.0)	0 (0.0)
Antibiotics administered	2 (100.0)	9 (90.0)
**Disease course, MD (IQR)**		
Duration of oxygen support in days	7.0 (2.0–12.0)	2.5 (2.0–4.0)
Duration of non-invasive ventilatory support in days	7.0 (7.0–7.0)	6.0 (3.0–9.0)
Duration of mechanical ventilation in days	N/A	1.0 (1.0–3.0)
Duration of hospital stay in days	11.5 (2.0–21.0)	3.5 (1.0–7.0)

(S)ARI – (severe) acute respiratory illness, CPAP – continuous positive airway pressure, HFNC – high-flow nasal cannula, IQR – interquartile range, MD – median, RSV – respiratory syncytial virus, SD – standard deviation, x̃ – mean.
*Values presented as n (%) unless specified otherwise.

Table S3**.** Demographics and clinical characteristics of RSV-positive inpatients included during and outside the study period*

	RSV-positive			Non-RSV (S)ARI		
	**During study period**	**Outside study period**	**P-value**	**During study period**	**Outside study period**	**P-value**
**Total number of participants**	469	17		177	83	
**Age in months, X̄ (SD)**	6.6 (5.6)	4.2 (5.2)†	0.14	6.5 (6.4)	8.6 (5.9)‡	0.05
**Female**	187 (39.9)	5 (29.4)	0.39	54 (30.5)	33 (39.8)	0.14
**Median household size, MD (IQR)**	4.84 (1.7)	5.8 (2.4)	0.05	4.8 (4.6)	5.4 (2.5)	0.05
**Prematurity**	29 (6.2)	1 (5.9)	0.98	19 (9.7)	6 (7.2)	0.64
**Comorbidity**	34 (7.2)	2 (11.8)	0.23	30 (17.0)	2 (2.4)	0.001
**Fully vaccinated according to local immunisation schedule and age**	445 (94.9)	16 (94.1)	0.85	163 (92.6)	81 (97.6)	0.23
**Maternal immunisation**	467 (99.6)	16 (94.1)	0.005	174 (98.9)	82 (98.8)	0.96
**Presentation upon admission**						
Time between onset of symptoms and hospital admission in days, X̄ (SD)	3.1 (1.6)	3.9 (1.6)	0.05	3.3 (2.0)	3.9 (3.6)	0.02
Fever (temperature >38ºC)	305 (65.0)	10 (58.8)	0.60	116 (65.5)	45 (54.2)	0.08
Oxygen saturation on room air, X̄ (SD)	88.4 (5.2)	85.3 (2.5)	0.04	89.9 (5.9)	89.9 (4.3)	0.96
Concomitant non-respiratory diagnosis						
**Disease management**						
Oxygen support	271 (57.8)	16 (94.1)	0.003	74 (41.8)	38 (45.8)	0.55

Table S3. Continued

Non-invasive ventilatory support (CPAP or HFNC)	131 (27.9)	10 (58.8)	0.006	31 (17.5)	12 (14.5)	0.54
Mechanical ventilation	1 (0.6)	0 (0.0)	0.75	9 (5.1)	1 (1.2)	0.13
Systemic corticosteroids administered	125 (26.7)	8 (47.1)	0.06	30 (17.0)	31 (37.4)	0.00
Antibiotics administered	425 (90.6)	15 (88.2)	0.74	149 (84.2)	78 (94.0)	0.03
**Disease course**						
Duration of oxygen support in days	2.0 (1.0–3.0)	2.0 (1.0–2.0)	0.68	2.0 (1.0–3.0)	2.0 (1.0–2.0)	0.18
Duration of non-invasive ventilatory support in days	2.0 (1.0–3.0)	2.0 (2.0–3.0)	0.14	2.0 (1.0–3.0)	2.0 (1.0–7.0)	0.23
Duration of mechanical ventilation in days	4.0 (3.0–6.0)	-	N/A	4.0 (1.0–11.0)	3.0 (3.0–3.0)	0.59
Duration of hospital stay in days	4.0 (3.0–6.0)	6.0 (4.0–9.0)	0.004	4.0 (3.0–7.0)	4.0 (4.0–6.0)	0.68

(S)ARI – (severe) acute respiratory illness, CPAP – continuous positive airway pressure, HFNC – high-flow nasal cannula, IQR – interquartile range, MD – median, RSV – respiratory syncytial virus, SD – standard deviation, $\tilde{x}$ – mean.
*Values presented as n (%) unless specified otherwise.
† & ‡ Indicate variables with more than 10% missing observations. Missing data are due to incomplete or missing clinical records. † n=13/17; ‡ n=47/83.

Table S4. Overview of costs (in 2023 USD) per LRTI episode for RSV-positive and non-RSV (severe) acute respiratory illness patients < 2 years old, comparing outpatients and inpatients

	**RSV positive (n=532)**		**RSV negative (n=198)**	
	**Outpatient (n=63)**	**Inpatient (n=469)**	**Outpatient (n=21)**	**Inpatient (n=167)**
**Societal costs total**				
X̄ (95% CI)	43.43 (24.77–62.09)	333.33 (306.05–360.60)	70.09 (20.68–119.51)	432.60 (374.48–490.73)
MD (IQR)	28.30 (24.68–39.46)	270.27 (180.57–408.35)	31.00 (24.88–47.82)	324.23 (205.73–469.28)
Direct medical costs				
X̄ (95% CI)	21.61 (20.28–22.94)	136.29 (125.83–146.75)	24.66 (20.11–29.22)	193.98 (160.59–227.36)
MD (IQR)	20.43 (18.92–23.99)	108.99 (84.17–150.28)	19.04 (18.03–26.11)	126.19 (90.02–200.83)
Direct non-medical costs				
X̄ (95% CI)	8.32 (3.00–13.65)	67.14 (57.58–76.72)	34.98 (0.00–81.46)	78.81 (65.19–92.42)
MD (IQR)	1.80 (0.84–5.83)	37.60 (20.46–69.56)	7.52 (0.90–13.99)	45.84 (21.79–105.66)
Indirect costs				
X̄ (95% CI)	13.50 (0.00–27.20)	132.49 (117.43–147.56)	10.46 (2.24–18.67)	160.92 (132.04–189.80)
MD (IQR)	3.40 (0.63–9.61)	95.70 (48.41–161.12)	2.27 (1.51–7.63)	111.54 (48.41–209.47)
**Health system costs total**				
X̄ (95% CI)	15.78 (15.04–16.52)	58.05 (53.86–80.87)	15.93 (15.26–16.60)	67.91 (59.12–76.71)

Table S4. Continued

MD (IQR)	14.71 (14.71–17.84)	49.08 (36.83–65.70)	14.71 (14.71–17.84)	58.09 (40.17–80.87)
**Household costs total**				
X̄ (95% CI)	18.92 (9.92–27.93)	216.71 (199.33–234.10)	49.86 (1.01–98.72)	293.63 (247.27–339.99)
MD (IQR)	11.00 (7.36–16.79)	171.99 (110.86–263.46)	13.15 (9.88–33.11)	207.82 (128.19–316.14)
Direct medical costs				
X̄ (95% CI)	5.83 (4.61–7.05)	78.25 (69.92–86.57)	8.73 (4.45–13.02)	126.06 (96.30–155.82)
MD (IQR)	5.23 (3.03–7.45)	55.74 (34.90–93.73)	4.14 (2.63–8.27)	71.08 (39.41–125.26)
Direct non-medical costs				
X̄ (95% CI)	8.32 (2.99–13.65)	67.14 (57.56–76.72)	34.98 (0.00–81.46)	78.81 (65.19–92.42)
MD (IQR)	1.80 (0.84–5.83)	37.60 (20.46–69.56)	7.52 (0.90–13.99)	45.84 (21.79–105.66)
Indirect costs				
X̄ (95% CI)	4.77 (1.23–8.32)	72.80 (67.58–78.03)	6.16 (1.50–10.81)	89.48 (76.58–102.37)
MD (IQR)	2.27 (0.44- 4.54)	60.70 (36.69–92.22)	2.27 (1.51–4.54)	66.57 (36.56–115.22)

CI - confidence interval, IQR - interquartile range, MD - median, RSV - respiratory syncytial virus, LRTI - lower respiratory tract infection, x̃ – mean

Table S5. Breakdown of direct medical costs components (in 2023 USD) per ARI episode, according to severity level

	RSV-positive (n=532)			Non-RSV (S)ARI (n=198)		
	**Non-severe (n=63)**	**Severe (n=441)**	**Life-threatened (n=28)**	**Non-severe (n=21)**	**Severe (n=149)**	**Life-threatened (n=28)**
**Medication costs**						
X̄ (95% CI)	2.57 (2.16–2.98)	13.67 (12.59–14.74)	35.52 (22.82–48.22)	2.89 (1.61–4.17)	16.16 (13.53–18.79)	55.64 (35.01–76.27)
MD (IQR)	2.45 (1.20–3.65)	11.01 (7.38–16.55)	19.53 (13.86–42.97)	2.14 (0.90–3.38)	11.14 (7.98–17.58)	35.37 (16.45–81.37)
Antibiotics						
X̄ (95% CI)	0.98 (0.74–1.23)	4.34 (3.76–4.91)	15.52 (6.86–24.18)	1.19 (0.90–1.48)	6.15 (4.26–8.05)	29.15 (13.25–45.05)
MD (IQR)	0.86 (0.61–1.20)	3.37 (2.34–4.70)	6.86 (3.05–13.46)	1.03 (0.95–1.35)	3.38 (2.20–5.08)	8.47 (5.19–43.38)
Steroids						
X̄ (95% CI)	0.86 (0.11–1.60)	2.94 (2.49–3.39)	3.92 (1.98–5.86)	0.45 (0.34–0.56)	4.10 (2.45–5.75)	5.09 (1.83–8.34)
MD (IQR)	0.86 (0.36–1.35)	2.29 (1.24–3.23)	3.78 (2.48–6.42)	0.45 (0.38–0.53)	2.48 (1.65–4.14)	4.55 (2.48–5.88)
Bronchodilators						
X̄ (95% CI)	0.86 (0.65–1.07)	3.91 (3.47–4.35)	7.47 (3.96–10.99)	1.09 (0.30–1.87)	3.45 (2.97–3.92)	2.95 (2.00 -3.90)
MD (IQR)	0.83 (0.38–1.05)	2.95 (1.70–4.51)	3.61 (2.71–8.12)	0.76 (0.45–1.40)	2.78 (1.68–4.27)	2.89 (1.71–4.28)
Other drugs						
X̄ (95% CI)	0.59 (0.41–0.77)	1.66 (1.43–1.89)	6.91 (3.50–10.32)	0.77 (0.48–1.05)	2.21 (1.61–2.82)	8.48 (4.17–12.79)
MD (IQR)	0.30 (0.23–0.83)	1.01 (0.47–1.97)	4.23 (1.85–6.97)	0.69 (0.42–1.17)	0.99 (0.47–2.25)	6.47 (2.79–9.85)

CI - confidence interval, (S)ARI – (severe) acute respiratory illness, IQR - interquartile range, MD - median, RSV - respiratory syncytial virus, LRTI - lower respiratory tract infection, x̃ – mean

RSV GOLD III – Health Economics Study Group members*

Cameroon:
Frédéric Debellut – Center for Vaccine Innovation and Access, PATH, Geneva, Switzerland
Norbert Fuhngwa – Triangle Research Foundation, Douala, Cameroon
Henshaw Mandi – Triangle Research Foundation, Douala, Cameroon

Ghana:
Rosemary Akuaku – Department of Child Health, Korle Bu Teaching Hospital, Accra, Ghana
Joycelyn Dame – University of Ghana Medical School Korle Bu Teaching Hospital, Accra, Ghana
Amma Ekem – Department of Child Health, Korle Bu Teaching Hospital, Accra, Ghana
Bamenla Goka – University of Ghana Medical School Korle Bu Teaching Hospital, Accra, Ghana
Ebenezer Ntow – Department of Child Health, Korle Bu Teaching Hospital, Accra, Ghana
Kwabena A. Osman – University of Ghana Medical School Korle Bu Teaching Hospital, Accra, Ghana

Mozambique:
Assucênio Chissaque – Instituto Nacional de Saúde, Marracuene district, Maputo, Mozambique; Instituto de Higiene e Medicina Tropical, Universidade Nova de Lisboa, Lisbon, Portugal
Nilsa de Deus – Instituto Nacional de Saúde, Marracuene district, Maputo, Mozambique
Esperança Lourenço Guimarães – Instituto Nacional de Saúde, Marracuene district, Maputo, Mozambique; Instituto de Higiene e Medicina Tropical, Universidade Nova de Lisboa, Lisbon, Portugal
Braiton Maculuve – Ministério da Saúde, Maputo, Mozambique
Elias Manjate – Faculty of Medicine, University Eduardo Mondlane, Maputo
Yara Manjate – Faculty of Medicine, University Eduardo Mondlane, Maputo
Izilda Matimbe – Faculty of Medicine, University Eduardo Mondlane, Maputo
Tufária Mussá – Faculty of Medicine, University Eduardo Mondlane, Maputo
Mirela Pale – Instituto Nacional de Saúde, Marracuene district, Maputo, Mozambique

Cesar Palha – Faculty of Medicine, University Eduardo Mondlane, Maputo
Cristina Sinussene – Faculty of Medicine, University Eduardo Mondlane, Maputo
Farida Zavala – Faculty of Medicine, University Eduardo Mondlane, Maputo

Nepal:
Ram H. Chapagain – Kanti Children's Hospital, Kathmandu, Nepal; Nepal Paediatrics Society, Kathmandu, Nepal
Rita Dhital - Nepal Paediatrics Society, Kathmandu, Nepal
Upendra Dhungana - Public Health Administrator; Ministry of Health and Population. Nepal
Prakash Joshi – Kanti Children's Hospital, Kathmandu, Nepal; Nepal Paediatrics Society, Kathmandu, Nepal
Ranju Karki - Nepal Paediatrics Society, Kathmandu, Nepal
Adita Nepali - Nepal Paediatrics Society, Kathmandu, Nepal
Uttam Paudel - Post Doctorate Researcher (Health Economics), Chulalongkorn University
Arun K. Sharma – Tribhuvan University Teaching Hospital, Institute of Medicine, Kathmandu, Nepal; Nepal Paediatrics Society, Kathmandu, Nepal
Rupesh Shrestha – Tribhuvan University Teaching Hospital, Institute of Medicine, Kathmandu, Nepal
Nirasta Thakili - Nepal Paediatrics Society, Kathmandu, Nepal

Nigeria:
Fadlulai Abdu-Raheem – Department of Paediatrics, Ahmadu Bello University Teaching Hospital, Zaria, Nigeria
Anas Abubakar – Department of Paediatrics, Ahmadu Bello University Teaching Hospital, Zaria, Nigeria
Abdullahi Aminu – Department of Paediatrics, Ahmadu Bello University Teaching Hospital, Zaria, Nigeria
Maria A. Garba – Department of Paediatrics, Ahmadu Bello University Teaching Hospital, Zaria, Nigeria
Fatima J. Giwa – Department of Medical Microbiology, Ahmadu Bello University Teaching Hospital, Zaria, Nigeria
Habiba Lawal – Institute of Child Health, Ahmadu Bello University, Banzazzau, Zaria
Bernsah D. Lawong – Department of Economics, Ahmadu Bello University, Zaria, Nigeria
Abdullahi Musa – Department of Paediatrics, Ahmadu Bello University Teaching Hospital, Zaria, Nigeria
Teddy Naddumba – Center for Vaccine Innovation and Access, PATH, Kampala, Uganda
Aira A. Olorukooba – Department of Paediatrics, Ahmadu Bello University Teaching Hospital, Zaria, Nigeria

Support:
Andrew Clark – Department of Health Services Research and Policy, London School of Hygiene & Tropical Medicine, London, UK
An Nguyen – Center for Vaccine Innovation and Access, PATH, Ho Chi Minh city, Vietnam
Clint Pecenka – Center for Vaccine Innovation and Access, PATH, Seattle, WA, USA

The Netherlands:
Louis J. Bont – University Medical Centre Utrecht, Utrecht, The Netherlands
Neele Rave – University Medical Centre Utrecht, Utrecht, The Netherlands
Farina L. Shaaban – University Medical Centre Utrecht, Utrecht, The Netherlands

* The authors are listed in alphabetical order of their surnames, according to the specific country teams with which they were involved in the study. Team members from University Medical Centre Utrecht, along with supporting staff, were involved at all study sites. A detailed overview of authorship contributions for each country can be found in the respective paper.
